# Simulation Approach for Hydrophobicity Replication via Injection Molding

**DOI:** 10.3390/polym13132069

**Published:** 2021-06-23

**Authors:** Tomás Baldi-Boleda, Ehsan Sadeghi, Carles Colominas, Andrés García-Granada

**Affiliations:** 1 Grup d’Enginyeria en Producte Industrial (GEPI), Institut Químic de Sarrià, Universitat Ramon Llull, Via Augusta 390, 08017 Barcelona, Spain; tomas.baldi@iqs.url.edu (T.B.-B.); ehsan.sadeghi@iqs.url.edu (E.S.); 2 Grup d’Enginyeria de Materials (GEMAT), Institut Químic de Sarrià, Universitat Ramon Llull, Via Augusta 390, 08017 Barcelona, Spain; carles.colominas@iqs.url.edu

**Keywords:** hydrophobicity, nanopattern, replication, plastic injection, simulation, computational fluid dynamics (CFD), finite elements (FE), volume of fluid (VOF), viscosity

## Abstract

Nanopattern replication of complex structures by plastic injection is a challenge that requires simulations to define the right processing parameters. Previous work managed to simulate replication for single cavities in 2D and 3D, showing high performance requirements of CPU to simulate periodic trenches in 2D. This paper presents two ways to approach the simulation of replication of complex 3D hydrophobic surfaces. The first approach is based on previous CFD Ansys Fluent and compared to FE based CFD Polyflow software for the analysis of laminar flows typical in polymer processing and glass forming as well as other applications. The results showed that Polyflow was able to reduce computing time from 72 h to only 5 min as desired in the project. Furthermore, simulations carried out with Polyflow showed that higher injection and mold temperature lead to better replication of hydrophobicity in agreement with the experiments. Polyflow simulations are proved to be a good tool to define process parameters such as temperature and cycle times for nanopattern replication.

## 1. Introduction

The use of nanotextured surfaces is increasing in many products due to their wide field of applications. Such surfaces are usually inspired from nature. Surface nanostructure include hydrophobicity [1], antireflectivity [2], antimicrobial properties [3], water treatment [4], lab-on-chip devices [5], etc. One of the best-known structures is the Lotus leaf effect [6] and there are several articles that intend to explain and copy the lotus effect [7]. However, creating a 10 mm × 10 mm sample with holes every 10 µm implies to manufacture 1000 × 1000 = 1 million holes, which remains nowadays expensive.

Injection molding is a low production cost manufacturing process [8] able to replicate many geometries due to the phase change of the molded material. Therefore, nanostructured surfaces are a possible target for this manufacturing process. However, it is challenging to copy the texture due to the solidification of the polymer before reaching 100% of the mold [9].

The purpose of this paper was to model and simulate replications of microstructures without the modelling of the full mold taking advantage of the periodicity of nanopattern. This approximation was made because the difference of magnitude order was large enough to make impossible the simulation even with extreme mesh refinement. Previous research by Pina et al. [10] used 2D simulations with Ansys Fluent to define which parameter of plastic injection was leading to a better replication. The title of research was “A statistical analysis of nanocavities replication applied to injection molding” because there was not a direct correlation between time in simulation or real replication depths. Using the same simulation approach, Pina-Estany and Garcia-Granada [11] showed that simulations are required to be made in 3D for low aspect ratios obtaining the same results as 2D simulations for long trenches to be replied. Finally, these researchers [12] studied replication for small cavities below 3 nm using 3D molecular dynamics approach with Large-scale Atomic/Molecular Massively Parallel Simulator (LA MMPS).

Parallel to this work, Biosca et al. [13] worked on the blow of glass bottles using the Ansys Polyflow finite elements (FE) approach. This study proved that viscosity was a key parameter to be correctly modelled as a function of temperature and shear rate. This work also showed that 2D axisymmetric simulations provided the same results as 3D simulations using less central processing unit (CPU) time for geometries where there is an axis of symmetry.

This study pretends to apply the viscosity dependence of temperature and shear rate for ANSYS Fluent simulations in order to check if time and depth are predicted not only as tendencies, but with real correlation. Furthermore, the simulation time for hydrophobic surfaces is reduced using 2D axisymmetry simplification of 3D structures. Moreover, Polyflow simulations are introduced for the first time for plastic injection in order to achieve a real simulation time decrease. All results are validated with experimental results presented by Baldi et al. [14].

## 2. Materials and Methods

### 2.1. Mathematical Formulation

In this section, the mathematical formulation of the Computational Fluid Dynamics (CFD) of the nanoplastic injection molding is discussed. For volume-of-fluid (VOF) simulations with heat transfer, the continuity, momentum, energy, and transport equations for each phase is solved. The so-called Navier–Stokes equations are a set of partial differential equations (PDEs) that generally do not have an analytical solution; therefore, numerical methods shall be applied to estimate the flow pattern in the domain of interest. In non-Newtonian shear-thinning fluids, the viscosity term depends on the temperature and shear rate of the fluid. In this study, the Cross-WLF (Williams–Landel–Ferry) viscosity model was used to capture the shear-thinning and cooling mechanism of the nanoplastic injection [15].

Equation (1) is the general form of the mass conservation equation, where *ρ* is the density, *t* is time, and V is the velocity vector of the flow. Equation (2) is the conservation of momentum in the x direction of the coordinate system, an inertial coordinate system. In this equation, V is the velocity vector, *u* the x-component of velocity vector, *p* the pressure, *f* body forces, and *F*_xv_ is the stress tensor which takes all the terms associated with viscosity. Energy equation (Equation (3)) relates the temperature field (*T*) and internal energy (*e*) to pressure and the rate of work done on the fluid by viscous effects ($\dot{Q}$) [16].

(1) $$\frac{\partial \rho }{\partial t}+\nabla \cdot \left(\rho V\right)=0$$

(2) $$\frac{\partial \left(\rho u\right)}{\partial t}+\nabla \cdot \left(\rho uV\right)=-\frac{\partial p}{\partial x}+\rho f_{x}+F_{xv}$$

(3) $$\frac{\partial }{\partial t}\left[\rho \left(e+\frac{V^{2}}{2}\right)\right]+\nabla \cdot \left[\rho \left(e+\frac{V^{2}}{2}\right)V\right]=\rho \dot{q-\nabla \cdot \left(pV\right)+\rho \left(f\cdot V\right)+\dot{Q_{v}}+\dot{W_{v}}}$$

Equations (1)–(3) are the strong form of Navier–Stokes equations that can be solved with various approaches such as finite elements (FE) or finite volume method (FVM). For the FVM, as it is used in ANSYS Fluent, the PDEs are discretized to many sub-domains called cells or volumes by using Gauss’ theorem. The weak form of the Navier–Stokes equations can be formulated in terms of the weak residual. The a posteriori error analysis can then be extended also to the Navier–Stokes equations, with modifications for the nonlinearity of the equations, and possible stabilization terms in the finite element method. Discrete values of field quantities such as pressure are stored in the cell centres and interpolated by different schemes depending on the desired order of accuracy. In this study, the spatial discretization of the plastic injection was achieved by using Second-Order Upwind Scheme for density and momentum. The staggering approach was used to pressure field discretization; the so-called Pressure Staggering Option (PRESTO) scheme gives more accurate results by calculating the pressure on cell faces. When solving the equations in a segregated manner, ANSYS FLUENT uses the phase coupled SIMPLE (PC-SIMPLE) algorithm [17] for the pressure–velocity coupling. PC-SIMPLE is an extension of the SIMPLE algorithm [18] to multiphase flows. The velocities are solved coupled by phases, but in a segregated fashion. The recursive property of the fixed-point algorithms represents, to second-order approximation, a power iteration with the matrix of Jacobian of the fixed-point function. Both explicit and implicit algorithms in ANSYS Fluent solver are particular instances of the fixed-point method [19].

Equation (4) relates viscosity at a given shear rate with viscosity at zero shear stress as a function of temperature. This is called the cross-WLF viscosity model.

(4) $$\mu =\frac{\mu_{0}}{1+{\left(\frac{\mu_{0}\dot{\gamma }}{\tau^{*}}\right)}^{1-n}}$$

where *μ* is the melt viscosity (Pa s), *μ*_0_ is the zero shear viscosity or the ‘Newtonian limit’ in which the viscosity approaches a constant at very low shear rates, $\dot{\gamma }$ is the shear rate (1/s), τ* is the critical stress (Pa) level at the transition to shear thinning, and *n* is the power law index.

The zero-shear viscosity is given by Equation (5):

(5) $$log_{10}\left(\mu_{0}\left(T\right)\right)=log_{10}(D_{1})+\left(-\frac{A_{1}\left(T-T^{*}\right)}{A_{2}+\left(T-T^{*}\right)}\right)$$

where *T* is the temperature (K), T* is the glass transition temperature, *D*_1_ (Pa s) and *A*_1_ (-), determined by curve fitting coefficients, and *A*_2_ (K) is calculated with Equation (6):

(6) $$A_{2}=A_{3}+D_{3}p$$

where *p* is the pressure (Pa), and *A*_3_ (K) and *D*_3_ (K/Pa) are data-fitted coefficients

Finally, the glass transition temperature is given by the Equation (7):

(7) $$T^{*}=D_{2}+D_{3}p\;$$

where *D*_2_ (K) is data-fitted coefficient.

For Polyflow simulations, the strong temperature dependence on polymer viscosity is commonly expressed using the VFT (Vogel–Fulcher–Tamman) equation [20], while [21] used a similar equation with parameter *A* in log scale.

(8) $$log_{10}\left(\mu \left(T\right)\right)=A+\frac{B}{T-T_{0}}$$

where *A* (log(Pa s)), *B* (K*log(Pa s)), and *T*_0_ (K) are empirical coefficients that must be defined for each polymer. By comparing Equations (5) and (6), we can conclude that the model is identical for zero shear rate where *μ* = *μ*_0_.

### 2.2. Mold Properties for Plastic Injection

**Figure 1:**
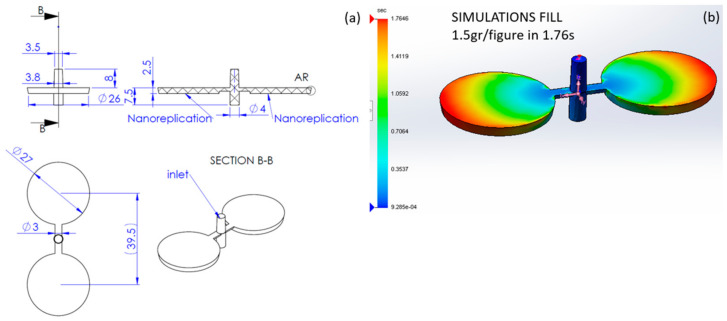
Plastic injection (**a**) basic dimensions and (**b**) macroscale simulation.

In order to understand the heat transfer between the polymer and the mold, we need to define the geometry of the part including channels and the core and cavity parts called mold. In this case, the mold is made of 1.2083 steel with relevant parameters for simulation shown in Table 1 comparing density *ρ*, thermal conductivity *α*, and specific heat *k*. The mold is polished to obtain a low friction and easy demolding. However, the lower circular surfaces of Figure 1 where nanopatterned using a femtosecond laser in order to obtain small cavities, which once correctly replicated, will lead to hydrophobic surface effect in the polymer.

In Figure 1, we observe that polymer injection is thought to obtain two small disc plates of around *d* = 26–27-mm diameter (with demolding angle) and 2.5-mm thickness. The volume of each plate is around 1.38 cm^3^ plus the volume of channels. The projected nanostructured area is around 2(π*d*^2^/4) ≈ 1100 mm^2^.

**Figure 2:**
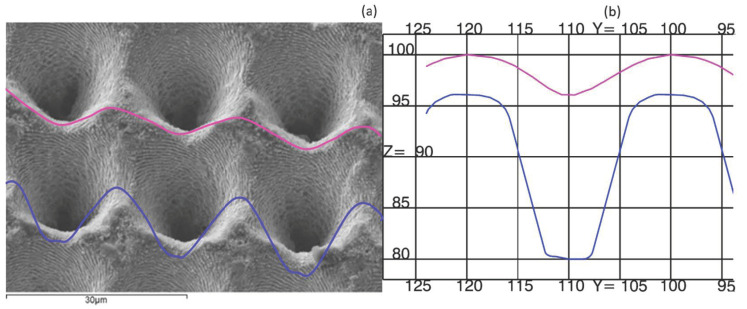
(**a**) SEM micrograph of micro-holes of the mold and (**b**) detailed CAD sections.

Injection molding is performed on a laser textured mold. The texture is an array of micro-holes with a 20-µm distance in both directions, as shown in Figure 2. Each hole is an inverted cone. The diameter of the cone is approximately 15 µm and the depth is around 27 µm. The mold has also a second texture of around less than a micron that will not be considered on the simulation. Two sections of the nanotextured area are shown in Figure 2b. For simulations of hydrophobicity, 2D axisymmetry might be considered using the section of maximum depth where the polymer will try to reach the bottom surface before it completely freezes.

### 2.3. Polymer Properties for Plastic Injections

Polypropylene (PP) is used for simulation with basic properties shown in Table 1 in order to be compared to steel mold. Thermal conductivity is larger in steel by two orders of magnitude which allows the quick cooling of polymer once it touches the surface of steel mold. On the other hand, specific heat is lower in steel by one order of magnitude.

The specific heat accumulated in polymers is transferred to cold mold reducing the temperature of polymer while it travels from the injection point flowing to fill the cavity of the mold. The viscous properties of the polymer are temperature-dependent. To obtain the values, a formula approximation must be done. In this case, Fulcher dependence is obtained as the most approximate formula to obtain a relation between temperature and viscosity.

**Figure 3:**
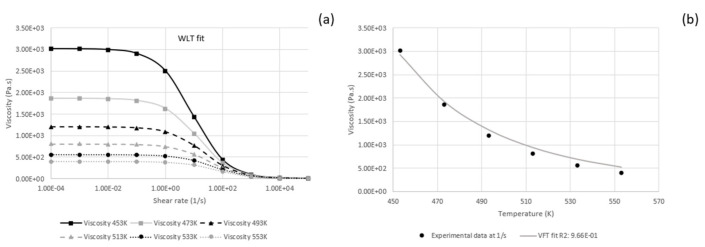
Viscosity (**a**) versus shear rate for different temperatures’ fitting WLF model and (**b**) curve fit for VFT model as a function of temperature for shear rates below 1(1/s).

Curve fitting of parameters lead to values expressed in Table 2 for the WLF model (used in Fluent) and shown in Figure 3a. Curve fitting for low shear rates was used to obtain the parameter in Table 3 and shown in Figure 3b for the VFT model (used in Polyflow).

### 2.4. Simulation Setup

All the simulations were performed with double-precision floating point accuracy and parallel processing capability in a workstation computer with Intel^®^ Xeon^®^ Gold 6230 Processor and 32 GB of RAM.

#### 2.4.1. SolidWorks Plastics Macro Simulation

The initial simulation considers the whole part including channels as shown in Figure 1. The plastic part was drawn in 3D using SolidWorks 2020 and the plastic injection simulation was carried out using WLF parameters from Table 1 for PP. The simulations considered melt temperature from 230 to 270 °C and mold temperature from 30 to 90 °C. The maximum injection pressure limit was set to 100 MPa with a maximum closing clamping force limit of 100 tn. With this setup, fill of the whole cavity was achieved in less than 2 s with a maximum required pressure of 4.2 MPa and clamping force of 2500 N (please note that 4.2 MPa × 1100 mm^2^ = 4620 N but pressure is not constant in all points at the same given time). Packing of the polymer was defined with a holding pressure and with a cooling time of up to 20 s each. This was done in order to decide when to open the mold and check if replication was complete. Obviously, this cooling time is required to be minimized in industry in order to maximize production and reduce cost per part.

All these values are shown in Supplementary Materials Figure S1, for a combination of melt and mold temperature. These values are obtained from conventional simulations carried out in industry as a previous step to nanoreplication.

#### 2.4.2. Fluent Approach for Nanoreplication

The initial attempt to simulate the plastic injection process was implemented in Ansys Fluent 2020 R2 software. Time-dependent multiphase model with pressure-based implicit and explicit formulation was used to simulate the two-phase flow model of plastic–air sharp interface. The volume of fluid (VOF) model includes the effect of surface tension along the interface between two phases; additionally, the contact angles between the phases (i.e., plastic–wall and air–wall contact) can be added. In computational fluid dynamics (CFD), transient simulations are generally computationally expensive in which the time-step and grid size play important roles in the accuracy and robustness of the simulations.

Owing to the physical geometry of the mold and expected periodic flow pattern in symmetry axis of the mold, an axisymmetric model was simulated and discretized in Ansys Design Modeler. The pressure inlet boundary condition was imposed with a value of 100 bar, and adjusted after the SolidWorks plastic injection simulations shown in Figure S1 (Supplementary Materials), and the no slip wall boundary condition was chosen for the exterior surfaces of the mold. Constant pressure inlet (Dirichlet boundary condition) was used to prescribe the polymer inlet in the entrance of the mold and zero Neumann boundary condition was imposed over the axis of symmetry of the mold.

**Figure 4:**
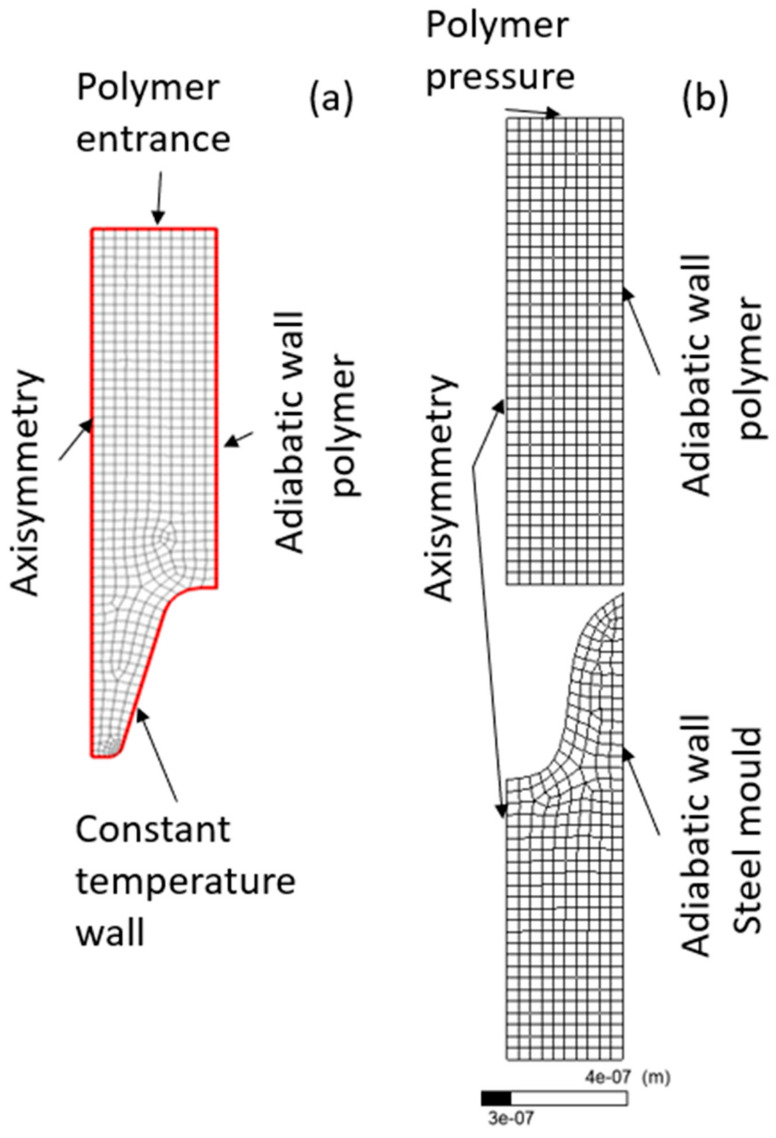
Mesh and boundary conditions used in (**a**) Fluent and (**b**) Polyflow.

The grid was generated by using linear elements with a total number of 3632 elements as shown in Figure 4. The linear elements, in contrast with quadratic elements, do not have midside nodes in the cell faces consequently reducing the number of global degrees of freedom and computational cost of the simulation. Triangles consist of three nodes plus one integration point and quads consist of four nodes plus one integration point. Quadratic elements capture the geometry curvature much better than the linear elements. The minimum edge length of the cells in the computational domain was in the order of 1 µm, which plays an important role in CFL (Courant–Friedrichs–Lewy) conditions and stability of the numerical scheme. In explicit schemes, the CFL or Courant number should be less than or equal to 1 in order to ensure the stability of the numerical scheme. The main difficulty of the nano-plastic injection process comes from the fact that, in order to meet the CFL criteria, the smaller time-steps in the order of 1 × 10^−15^ s should be taken into the account to accurately capture the plastic–air interface in the injection process. Parameters used for mesh generation included a growth rate of 1.2 capturing curvature and with smooth transition ratio of 0.25.

The ideal-gas density assumption for the air phase and constant density for the plastic phase was considered. The ideal-gas density law takes the compressibility of the trapped air inside the mold into account [22]. The viscous properties of the plastic follow a Cross-WLF model which describes the temperature, shear-rate, and pressure dependency of the viscosity. The Cross-WLF parameters of a generic PP are presented in Table 2. User-defined functions (UDF) were written for the viscosity model and adaptive time-step change and compiled in the fluent solver.

#### 2.4.3. Polyflow Approach for Nanoreplication

Ansys Polyflow 2020 R2 model was used to define the simplified simulation. The 2D axisymmetric model was used to avoid the 3D model and to reduce the number of nodes of the mesh. 2D geometry was imported from Solid Works model. Polyflow also allowed taking into account the changes in temperature and the variation of parameters such as viscosity with temperature, key to model solidification of the polymer.

The initial step of the simulations supposes that the polymer is already on top of the micro-hole and then the pressure of the injection pushed the polymer inside the micro-hole, as seen in previous group work [10]. Pressure was applied until the end of the cycle was at 15 s.

A standard quadrilateral mesh of 0.001-mm element size was used ensuring a minimum of 10 elements per edge resulting in an 813-node mesh as shown in Figure 4. The mesh was as the model, 2D. A local Lagrangian remeshing was added to the polymer in each iteration of the solver.

## 3. Results

Simulation times were dramatically reduced using the Polyflow approach by almost two orders of magnitude. Table 4 shows the reason for such reduction due to the requirements of small time-step increment in Ansys Fluent. Now with Polyflow, it is possible to provide results in 10 min, while with Fluent, only one result was provided every 12 h. It should be noted that SolidWorks simulations are required as boundary conditions for either Fluent or Polyflow simulations of nanoreplication; thus, such simulation time should be added when changing melt or mold temperatures.

### 3.1. Fluent

**Figure 5:**
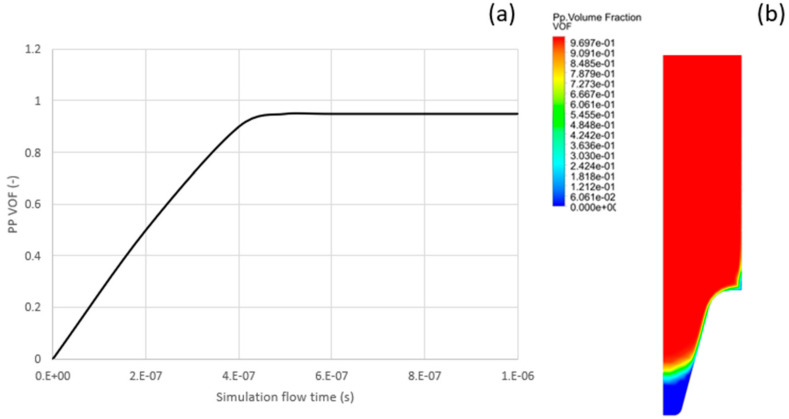
Fluent results showing (**a**) VOF versus time, and (**b**) VOF at end of simulation.

Figure 5b show the flow PP flow pattern at the end of injection time. As seen from the figure, a narrow air gap exists at the end of the mold. This air gap chokes the PP flow and prevents the plastic to be advanced further. The second graphics shows the flow front advancement versus flow time. The straight horizontal segment of the graph signifies that the plastic cannot be advanced due to the air gap and the high viscosity of the plastic as it cools down in the cavity.

The average computational time for this 2D-Axisymmetric simulation was approximately 12 h. 3D symmetric simulations were also performed in order to investigate the full-scale geometry effect on the heat loss of the plastic; however, the main difficulty with these simulations is their computationally expensive nature. A single 3D CFD simulation of the nano plastic injection molding was estimated at approximately 72 to 120 h (depending on the initial plastic temperature), which is a main drawback to robustly analyze the plastic–air interface and set full factorial design simulations.

Although the Fluent simulations were able to capture the advancing front of the polymer melt, the flow time was not in accordance with the experimental data. According to the fluent simulations, with the polymer temperature of 230 and mold temperature of 90, the plastic fills 97% of the mold; however, the total flow time is less than 2 × 10^−6^ s [14].

The fluent VOF approach with temperature and shear rate dependence of viscosity is able to capture the flow front trend, but further investigations shall be done to consider the local heat transfer, variable conduction heat transfer, and including effects of convection heat transfer.

### 3.2. Polyflow

**Figure 6:**
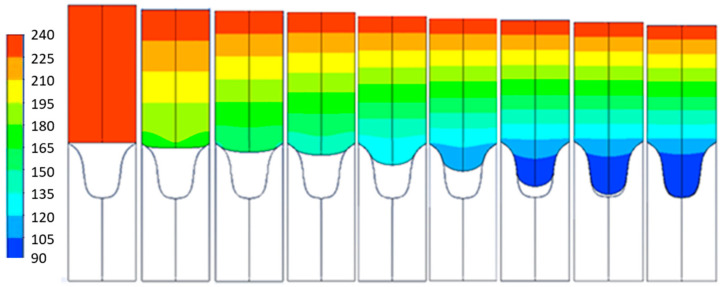
Evolution of Polyflow replication for T = 230 °C and Tm = 90 °C at different times from left to right of *t* = 0, 0.01, 0.05, 0.1, 0.5, 1, 5, 10, 15 s.

Several simulations have been carried out at different polymer and mold temperatures. In the simulations, one can observe the time evolution of the plastic to the mold and how it cools down as it enters the mold. As expected, the plastic gains viscosity as it cools and therefore, it advances more slowly towards the inside of the mold. The conditions of 230 °C plastic temperature and 90 °C mold temperature are shown in Figure 6. It can be seen how, at the end, the simulation is able to copy 100% of the mold geometry.

**Figure 7:**
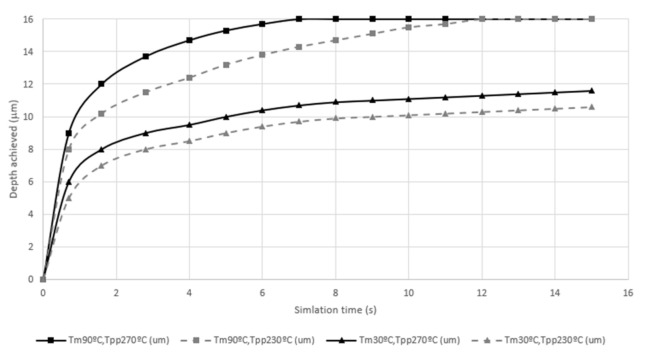
Y coordinate evolution of the bottom-middle corner point on different conditions vs. time (s).

All the results in different samples can be seen with the lower center point marked and the time evolution plotted in Y in Figure 7. With this approach, the evolution of all the samples can be seen and compared. Some of the samples do not manage to copy 100% of the mold after 15 s; thus, it is considered that the time required is sufficiently long to consider that, given the asymptotic behavior of the time evolution, they will not manage to copy or, at least, that they will not manage to copy within a reasonable time for a plastic injection molding copying process at an industrial level. Looking in more detail at the samples that do not copy, it can be seen that they are the ones that have a cold mold, 30 °C. The 90 °C samples, on the other hand, are considered to copy 100%, following what was already discovered by Muntada et al.’s study [9] in which it was predicted that the mold temperature has a great effect. One of the samples that copied 100% is at 8 s and the other at 11.5 s. Therefore, it can also be concluded that time is also a factor affecting the copying as demonstrated by Baldi et al. [14].

**Figure 8:**
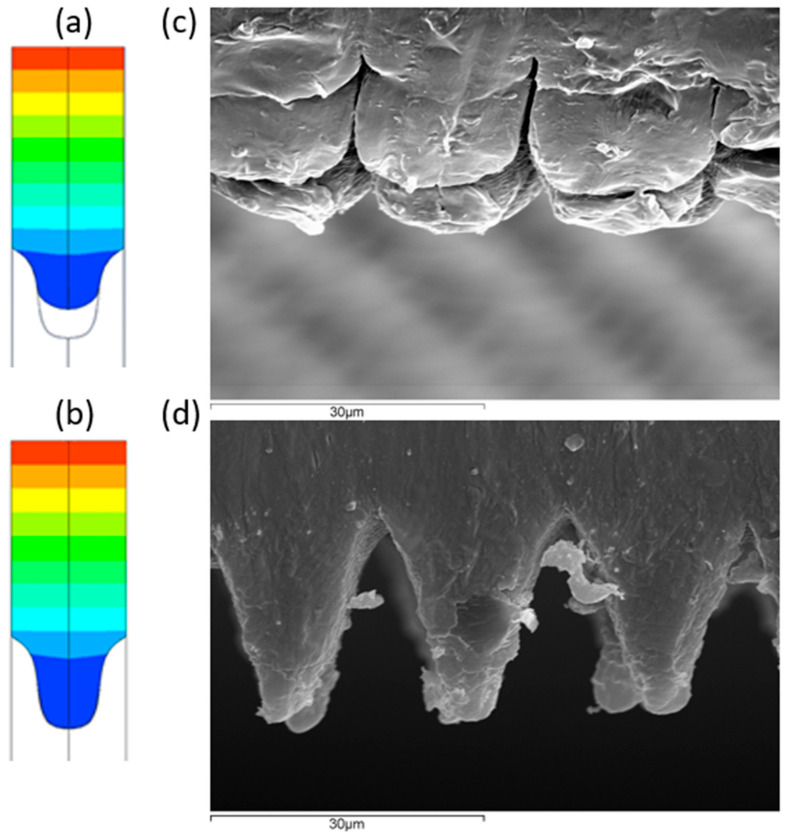
Simulation at ending time (15 s) on conditions (**a**) T = 230 °C, Tm = 30 °C and (**b**) T = 270 °C, Tm = 90 °C and SEM micrograph (**c**) T = 230 °C, Tm = 30 °C and (**d**) T = 270 °C, Tm = 90 °C.

To check whether the simulation results are correct or not, a copy and a section of the copied sample were analyzed and compared with the results obtained from the simulation. Figure 8 shows how, for the samples with the mold and cold plastic, a bad copy is obtained, very similar to that obtained in reality, of approximately 50% of the copied surface, while, with the hot mold, the copy is 100% of the mold surface. This is of vital importance as, if mass production is defined with injection parameters of cold mold, then the surface will not obtain hydrophobic properties as desired.

## 4. Discussion

**Figure 9:**
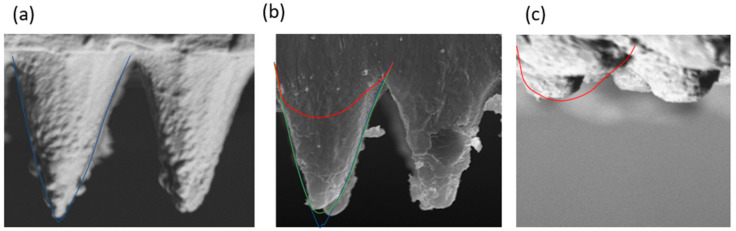
Experimental results of (**a**) best replication in silicone, (**b**) acceptable replication in PP with hot mold, and (**c**) incomplete replication in PP with cold mold.

Plastic injection is suitable for replication of hydrophobic surfaces using PP and the right combination of melt and mold temperature. Figure 9 shows the best replication of hydrophobic surface using silicone, a good hydrophobic copy using plastic injection with hot mold and a bad replication using a cold mold.

The measurements’ real replication height and injection time are in agreement with simulations which allow the designer to predict time and cost of production using simulation.

## 5. Conclusions

It is concluded that the Polyflow and Fluent approaches are valid for simulating how micro-holes are filled in the injection molding process. Ansys Fluent’s VOF approach was able to capture the advancing front of the polymer; however, the total flow time was not in accordance with the experimental data. Even though both approaches are valid, a simplified Polyflow model is less computationally expensive, as the calculation time is reduced to just 10 min.

The Polyflow model predicts the evolution of plastic for all the factors interfering on injection molding, such as temperature of the polymer, temperature of the mold, pressure, and filling time. The total time to obtain a full copy, key in the injection molding manufacturing process, can be predicted for each condition to optimize the production process.

Temperature of the plastic, temperature of the mold, time, and pressure are proportional to the copied percentage.

The results of the simulation are in agreement with those obtained in experimental studies.

## Figures and Tables

**Table 1:** Steel mold and PP polymer thermal material properties used in simulations.

	*ρ* (kg/m^3^)	α (W/(m K))	*k* (J/kg K)
Steel mold	7800	20	460
PP polymer	850	0.15	3100

Where *ρ* is density, *α* is thermal conductivity and *k* is specific heat.

**Table 2:** WLF parameters for viscosity as function of temperature for any shear rates.

*D1* (Pa·s)	*D2* (K)	*D3*^1^ (K/Pa)	*A1* (-)	*A3* (K)	*τ* (Pa)	*n* (-)	T*^1^ (K)	*A2*^1^ (K)
7.4 × 10^31^	113.15	0	32.7	51.6	26,260	0.272	113.15	51.6

^1^ With data provided, *D3* is zero and therefore, T* and *A2* are constant and do not depend on pressure.

**Table 3:** VFT parameters for viscosity as function of temperature for shear rates below 1(1/s).

*A*^1^ (log(Pa s))	*B*^1^ (K*log(Pa s))	*T*_0_ (K)
0.2	1110	113.15

^1^ A should be 0.2 + 3 in order to get viscosity in (cPo) instead of (Pa s) in Polyflow.

**Table 4:** Simulation times for Ansys Fluent versus Ansys Polyflow approach.

	CPU Time (s)	Simulated Time (s)	Mesh Size (mm)	Nodes (-)	Time Step (s)	Hard Disk Required (MB)
SolidWorks Plastics	1320	50	1	10,500	0.012 ^1^	93
Ansys Fluent	43,200	1 × 10^−6^	0.001	1092	1 × 10^−15^	80,000 ^2^
Ansys Polyflow	600	15	0.001	813	5 × 10^−3^	534

^1^ For SolidWorks Flow time step is 0.012 and for Pack, it is 0.5 s. ^2^ Writing only one out of 1-million-time steps.

## Data Availability

The data presented in this study are available in https://www.meaagg.com/PLASTFUN/PLASTFUN.html (accessed on 23 June 2021).

## Supplementary Materials

The following are available online at https://www.mdpi.com/article/10.3390/polym13132069/s1, Figure S1: Pressure and temperature as a function of time during flow and pack at 4 different locations, inlet pressure and clamping force as function of time in macrosimulation., Figure S1: Shear rate at time 0.002067 s, 0.2164 s, 1.645 s and 15 s to show that there is no shear rate as the cavity is completely filled.
