# Supplementary material for: Simulation Approach for Hydrophobicity Replication via Injection Molding
Source: Polymers (Basel). 2021 Jun 23;13(13):2069. doi: 10.3390/polym13132069 (PMC8272097; doi:10.3390/polym13132069)
Supplement: Supplementary file 1 [file polymers-13-02069-s001.zip › polymers-1267648-supplementary-for conversion.pdf]

polymers

MDPI

Supplementary Materials

Simulation Approach for Hydrophobicity Replication via Injection Molding

Tomás Baldi-Boleda [1], Ehsan Sadeghi [1], Carles Colominas [2] and Andrés García-Granada [1,*]

[1] Grup d'Enginyeria en Producte Industrial, (GEPI), Institut Químic de Sarrià, Universitat Ramon Llull, Via Augusta 390, 08017 Barcelona, Spain; tomas.baldi@iqs.url.edu (T.B.); ehsan.sadeghi@iqs.url.edu (E.S.); andres.garcia@iqs.url.edu (A.G.)

[2] Grup d'Enginyeria de Materials, (GEMAT), Institut Químic de Sarrià, Universitat Ramon Llull, Via Augusta 390, 08017 Barcelona, Spain; carles.colominas@iqs.url.edu (C.C.)

* Correspondence: andres.garcia@iqs.url.edu; Tel.: +34 932 672 083

Pressure and temperature as a function of time during flow and pack are provided at 4 different locations as input data to be used as boundary conditions in Polyflow simulations

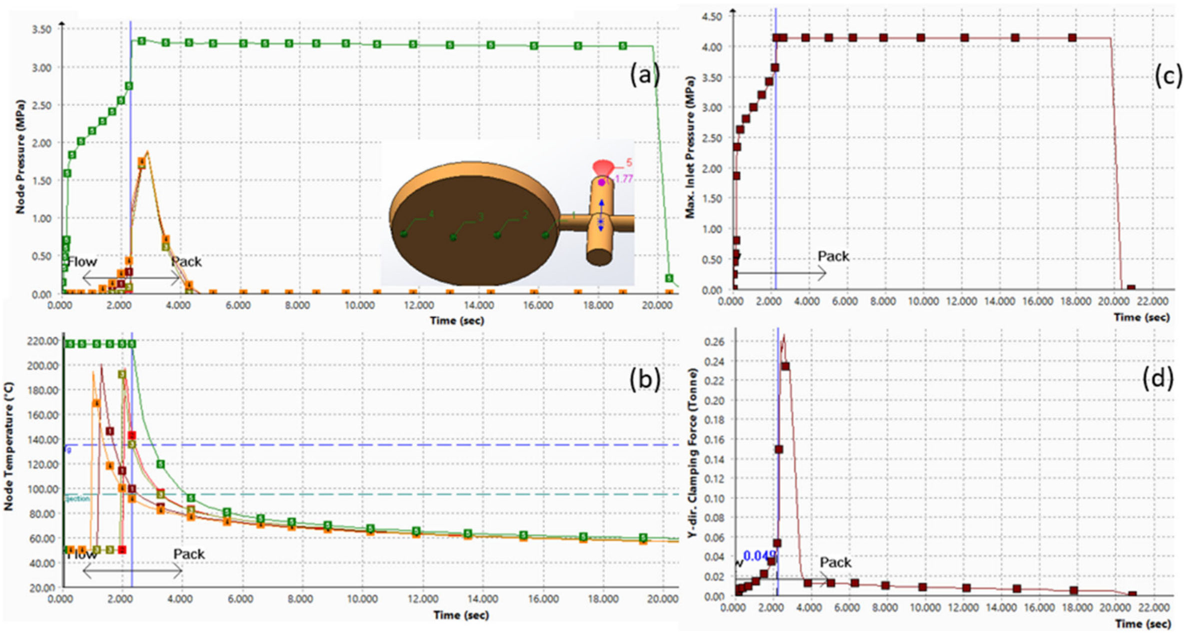

Figure S1. (a) Pressure and (b) temperature as a function of time during flow and pack at 4 different locations, (c) inlet pressure and (d) clamping force as function of time in macrosimulation.

During polyflow simulations shear rate was checked to be below 1s-1 to check the correct assumptions of using simplified VFT model in polyflow

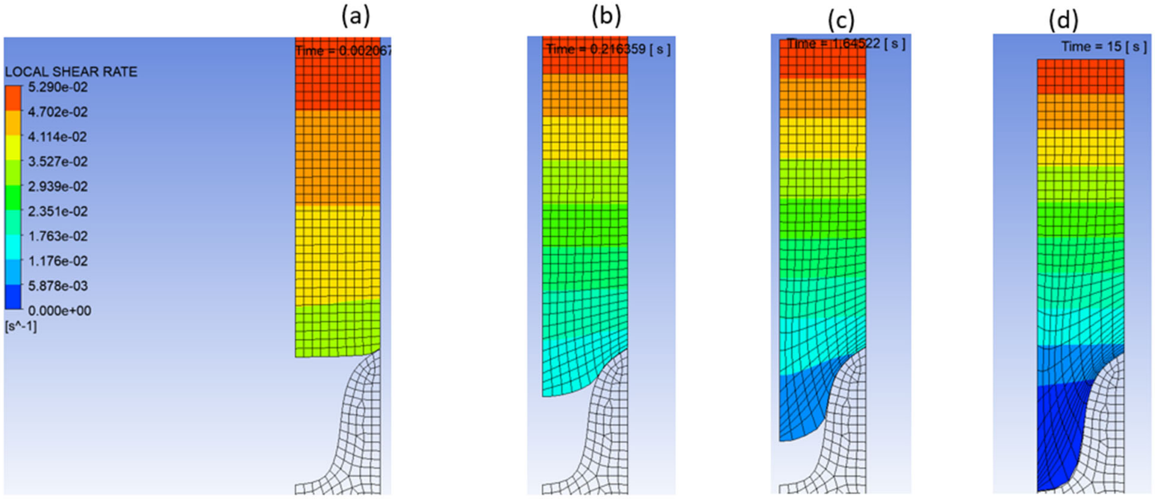

Figure S2. Shear rate at time (a) 0.002067s (b) 0.2164s (c) 1.645s and (d) 15s to show that there is no shear rate as the cavity is completely filled.
